# Herd level economic comparison between the shape of the lactation curve and 305 d milk production

**DOI:** 10.3389/fvets.2022.997962

**Published:** 2022-09-15

**Authors:** Yongyan Chen, Miel Hostens, Mirjam Nielen, Jim Ehrlich, Wilma Steeneveld

**Affiliations:** ^1^Department of Population Health Sciences, Faculty of Veterinary Medicine, Utrecht University, Utrecht, Netherlands; ^2^Dairy Veterinarians Group, Argyle, NY, United States

**Keywords:** lactation curve, milk production, dairy, economics, herd aggregation

## Abstract

Herd milk production performance is generally evaluated using the herd's average 305-day milk production (HM305). Economic comparisons between herds are also often made using HM305. Comparing herds is thus based on summarized milk production, and not on the form of the lactation curves of the cows within the herd. Cow lactation curve characteristics can be aggregated on a calendar year basis to herd lactation curve characteristics (HLCC) (herd magnitude, herd time to peak yield and herd persistency). Thus far, no literature has evaluated whether the shape of the lactation curve (described by HLCC) is better able to explain the economic variation of herds than summarized milk production such as HM305 does. This study aims to determine whether HM305 or HLCC is better able to explain the variation in economic performance between herds. To do so, we evaluated 8 years of Dutch longitudinal data on milk production and the financial accounts of 1,664 herds. Cow lactation curve characteristics were calculated through lactation curve modeling and aggregated to HLCC on a calendar year basis for two parity groups (primiparous cows and multiparous cows). Using income over feed cost per cow (IOFC-cow) or per 100 kg milk (IOFC-milk) as the dependent variable separately, we developed four linear mixed models. Two models were used to analyse the association between herd economic performance and HLCC; the other two models were used to analyse the association between herd economic performance and HM305. A Cox test and J test were used to compare two non-nested models to investigate whether HM305 or HLCC better explain IOFC. The average IOFC-cow was €2,305 (SD = 408) per year, while the average IOFC-milk was €32.1 (SD = 4.6). Results showed that HLCC and HM305 explain the same amount of variance of IOFC-cow or IOFC-milk. IOFC-cow was associated with HM305 and HLCC (except herd time to peak yield for primiparous cows). Herd magnitude was most strongly associated with IOFC-cow, followed by herd persistency and herd time to peak yield of multiparous cows. IOFC-milk was not associated with HM305 or HLCC (except for a weak negative association with herd persistency for primiparous cows). IOFC-cow and IOFC-milk were driven most by time effects. In conclusion, HLCC and HM305 explain the same amount of variance in IOFC-cow or IOFC-milk. HLCC is more computationally expensive, while HM305 is more readily available.

## Introduction

The milk production performance of cows is generally described by 305-d milk production (M305) (1, 2), which is an indicator of absolute milk production. Additionally, lactation curve characteristics (LCC), describing the lactation curve in different ways, can also be used to evaluate the milk production performance of cows. LCCs are derived from a lactation curve model such as the classic Wood model (3), the Wilmink model (4) and the Milkbot model (5). The MilkBot model, for example, consists of the scale (representing the level of production), the ramp (representing the rising rate of milk production up to the peak level), the estimated time between the start of milk synthesis and calving (offset) and the rate of late lactation decline (decay). The latter can be easily transformed into a measure of persistency (5). Both M305 and LCC are commonly used to compare the milk production performance of cows (6, 7) as well as economic performance (8, 9). Results show that cows with a higher M305 have lower costs per kg of milk and produce a higher IOFC-cow (8).

The milk production performance of the herd is generally evaluated using the herd's average 305-day milk production (HM305) (10–12) along with some other variables such as average milk production per cow per year (13, 14). Economic comparisons between herds are also generally made using HM305 (corrected for milk price) (15, 16). Comparing herds is thus based on the absolute volume of milk production rather than on the form of the lactation curves of the cows within the herd. Comparing herds based on LCC is challenging as LCCs are at cow level. Chen et al. (17) have already presented a procedure to aggregate the individual cow level LCC to the annual herd level for primiparous and multiparous cows separately. The annual herd lactation curve characteristics (HLCC) open possibilities to explore differences between herds. Potentially, HLCC can be an additional herd performance indicator. It differs between herds since the environment, management and cow genetics of a dairy herd influence individual cow's LCCs and hence HLCCs (18, 19). Persistency is one of the lactation curve characteristics that was shown to increase profitability at cow level, where more persistent cows were more profitable (8, 20, 21). This association was not studied at herd level, where HLCC might be associated with herd level economic results. It is therefore not known whether the shape of the curve is better or worse than the absolute volume of milk production at explaining the economic variation of herds. The herd's economic performance can be expressed in many ways, depending on data availability and the aim of the research. A herd's economic performance includes revenues, fixed costs and variable costs, which are difficult data to gather precisely. When the value of farm assets is not well-known, partial measure of farm profitability can be used (14, 22), such as gross margin, income over feed cost and milk-to-feed price ratio. Gross margin states the difference between total revenues and total variable costs. If only milk revenue and feed costs data are available, economic calculations, such as income over feed cost and milk-to-feed price ratio, can be used (23–25). Income over feed cost is often used to monitor whether the feed cost is in line for the milk production or whether the feed management is successful (26–28). Milk-to-feed price ratio indicates the convenience of transforming feed into milk in terms of market opportunity (25). However, when the price of milk and feed are volatile income over feed cost is a better measure of profitability than milk-to-feed price (23).

This study aims to determine whether HM305 or HLCC is better at explaining the economic performance variation between herds.

## Materials and methods

### Available data

For this study, we obtained milk production data at the test-day level and herd level performance data for the years 2007–2016 from the Dutch Cattle Improvement Cooperative (CRV, Arnhem, The Netherlands). Originally, the cow test-day data included 159,173,868 test-day records from 6,710,117 cows in 20,760 herds. All test-day records included general cow information (e.g., birth date, calving date, parity, health status), milk yield (kg) and milk component (protein and fat percentage). At the cow level, days in milk, age in days and calving intervals were calculated for every lactation. Herd level performance data contained annual averages of somatic cell counts (SCC), calving intervals, age in days and HM305.

We retrieved herd accounting data from a Dutch accounting agency (Flynth, Arnhem, the Netherlands). The data represented 2,058 commercial herds with 18,108 yearly records from 2008 to 2015, herd size varied between 5 and 1,075. The herd accounting data included annual information on all revenues (e.g., milk, livestock), fixed costs (e.g., depreciation, maintenance costs) and variable costs (e.g., feed costs, breeding costs, health costs), as well as on general herd characteristics (e.g., soil type, herd size, milking system).

### Development of HLCC

The development of HLCC was described in detail in our previous study (17). In short, we used the cow test-day data to calculate HLCC. First, we fitted a lactation curve for each whole lactation with the MilkBot model using a proprietary maximum likelihood fitting algorithm of the DairySight fitting engine (5). The full MilkBot equation is shown as:


(1) $$\begin{array}{l} Y\left(t\right)\;=\;a\left(1\;-\;\frac{e^{\frac{c\;-\;t}{b}}}{2}\right)e^{-dt} \end{array}$$

in which Y(t) is the estimated milk production when days in milk is t, and a (scale), b (ramp), c (offset), and d (decay) are LCC describing the lactation curve. As offset is practically undetectable without daily milk production records at the beginning of lactation, we decided not to use that measurement, resulting in a simplified equation:


(2) $$\begin{array}{l} Y\left(t\right)\;=\;a\left(1\;-\;\frac{e^{\frac{-t}{b}}}{2}\right)e^{-dt} \end{array}$$

In the current study, a (scale) was renamed magnitude of milk production (in kg/day) and b (ramp) was renamed time to peak yield (in days). d (decay) was transformed into a measure of persistency using the Equation (5):


(3) $$\begin{array}{l} \text{Persistency }=\;\frac{0.693}{\text{d}} \end{array}$$

Persistency (in days) is the time needed for milk production to drop by half after the peak.

After fitting, every lactation had a set of three LCCs (magnitude, time to peak yield and persistency). Two parity groups were defined: primiparous cows and multiparous cows. To summarize HLCC on a calendar year basis, we used a weighted method (17) as the partitioning method to deal with lactations in multiple calendar years. Lactations belong to every calendar year with a specific weight relative to the number of test-day records. Using the number of test-days as weight, the contribution of the lactation for different calendar years was calculated. For example, cow A started a lactation in 2008 and finished in 2009. This lactation had 5 test-day records in 2008 and 3 in 2009. Suppose there were n and m test-day records in total from all lactations in 2008 and 2009 in the herd. Cow A's lactation curve characteristics would contribute 5/n to the herd lactation curve characteristics in 2008 and 3/m in 2009. Using the number of test days per year as weight, the median HLCCs were defined as the annual HLCC per parity group, per herd and per year. As described in Chen et al. (17), we only included complete lactations to aggregate at herd level and thus excluded herd level calculations for the first record year (2007) and last record year (2016), resulting in 273,322 records from 20,000 herds. The lactation lengths varied between 56 (5%) and 495 (95%) days, with a mean of 336 days.

### Data management

The definition of all variables is shown in Table 1. We defined several additional variables based on the accounting dataset. First, income over feed cost (IOFC) was calculated as total milk revenue minus total feed costs (23, 30). Total annual milk revenue was available in the dataset and total annual feed costs were calculated by adding up the annual costs for concentrates, vitamins, minerals, wet by-products and roughage. We calculated two variables for IOFC, one expressed per cow (IOFC-cow) and the other expressed per 100 kg milk (IOFC-milk).

**Table 1:** Descriptive statistics of continuous variables over 1,664 Dutch herds for the years 2008–2015.

	**Description (unit)**	**Mean**	**SD**	**5%^a^**	**95%^a^**
IOFC-cow^b^	(Milk revenue – feed cost)/herd size (€)	2,305	408	1,609	2,961
IOFC-milk^c^	100*(Milk revenue – feed cost)/milk delivered to factory (€)	32.1	4.6	24.0	39.3
HM305	Average 305-day milk production in the herd (kg)	8,686	899	7,099	10,107
Equity ratio	(Total assets – total liabilities)/total assets	0.45	0.31	−0.11	0.93
Herd intensity	Milk production per ha (kg of milk/ha)	15,129	3,841	9,564	22,159
Relative herd milk price	The price difference in relation to national raw milk price^d^ (€/100 kg)	2.52	2.08	−0.86	5.71
Herd size	Number of cows present in the herd	85.3	43.2	39.0	151.0
Expansion rate	((herd size – last year's herd size)/last year herd size)/year difference	0.03	0.06	−0.06	0.14
Age in days	Average age in days of cows in the herd	1,716	160	1,483	1,998
Somatic cell counts	Average somatic cell counts of cows in the herd (*10^3^ cells/ml)	193	59	105	301
Calving interval	Average calving interval of cows in the herd	414	23	384	457
Herd magnitude1^e^	Weighted median magnitude of primiparous cows (kg/day)	34.8	3.7	28.3	40.5
Herd time to peak yield1	Weighted median time to peak yield of primiparous cows (day)	29.6	0.4	28.9	30.2
Herd persistency1	Weighted median persistency of primiparous cows (day)	358	70	263	492
Herd magnitude2+^f^	Weighted median magnitude of multiparous cows (kg/day)	47.7	5.3	38.0	55.8
Herd time to peak yield2+	Weighted median time to peak yield of multiparous cows (day)	22.1	1.3	20.3	23.3
Herd persistency2+	Weighted median persistency of multiparous cows (day)	240	33	194	304

^a^ 5% and 95%: the 5% and 95% percentile.

^b^ IOFC-cow: income over feed cost per cow.

^c^ IOFC-milk: income over feed cost per 100 kg.

^d^ Average yearly raw milk price aggregated by monthly raw milk price from official milk market observatory (29).

^e^ 1: primiparous cows.

^f^ 2+: multiparous cows.

Secondly, we calculated annual herd milk prices by dividing the total kg of milk delivered to the factory by milk revenue. In addition, we looked up average Dutch yearly raw milk prices (29) and calculated the relative annual herd milk price as the difference between herd milk price and the Dutch raw milk price for the corresponding year. Thirdly, as an indication of the level of economic leverage, we calculated the equity ratio per herd per year as follows:


$$\begin{array}{l} \text{Equity ratio }=\frac{\left(\text{total assets }-\text{ total liabilities}\right)}{\text{total assets}} \end{array}$$

Finally, we calculated the expansion rate from year n to year m as follows:


$$\begin{array}{l} \text{Expansion rate }=\;\frac{\left[\frac{\left(\text{herd size in m year}-\text{ herd size in n year}\right)\;}{\text{herd size in n year}}\right]}{\left(\text{m}-\text{n}\right)} \end{array}$$

**Figure 1:**
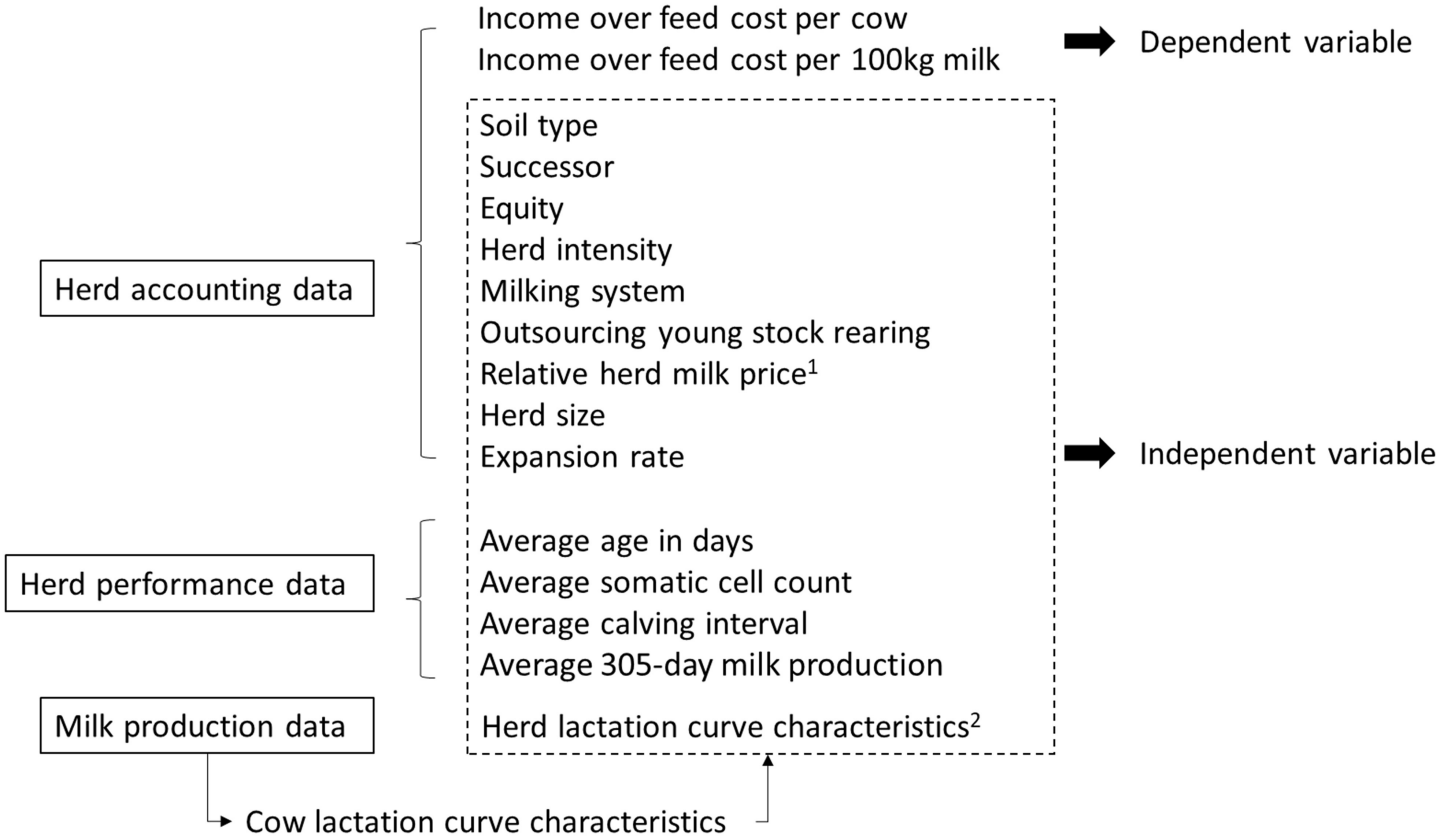
Overview of variables used in the statistical analyses and the dataset they originate from. ^1^the difference in milk price and the Dutch raw milk price for the corresponding year. ^2^herd magnitude, herd time to peak yield and herd persistency for primiparous cows and multiparous cows (17).

An overview of all datasets and defined variables included is shown in Figure 1.

The yearly herd accounting data of 2,058 herds were merged with calculated HLCC (*n* = 20,000 herds) and herd performance data (*n* = 20,760 herds) for the corresponding years. This merging was possible for 1,887 herds and resulted in a dataset of 12,849 yearly records from 2008 to 2015 for further analysis.

**Figure 2:**
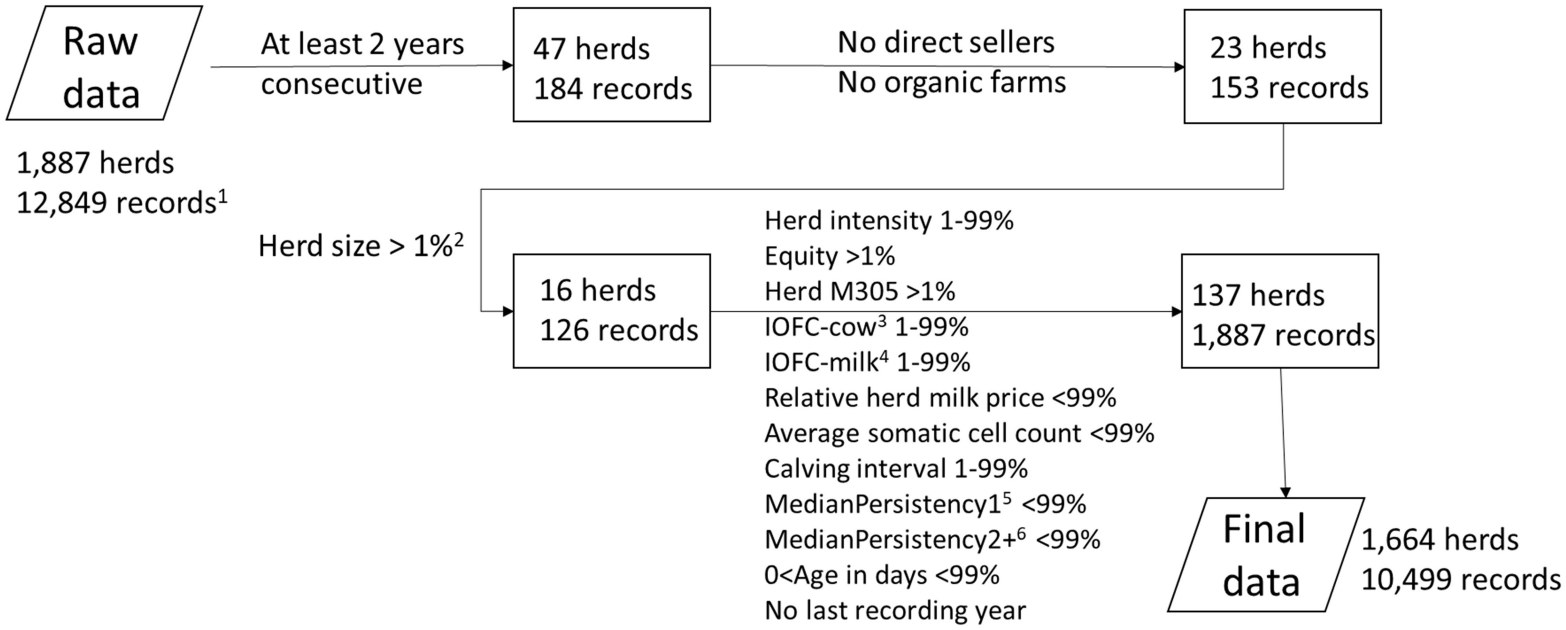
Diagram on data editing of the combined production and accounting dataset. The numbers in the boxes represent the excluded numbers. ^1^yearly records. ^2^%: percentile. ^3^IOFC-cow: income over feed cost per cow. ^4^IOFC-milk: income over feed cost per 100 kg milk. ^5^1: for primiparous cows. ^6^2+: for multiparous cows.

The data editing flow diagram is presented in Figure 2. We first excluded 184 yearly records as they were not consecutive (<2 years consecutive). Secondly, we excluded herds selling milk products on farms (direct sellers) and organic herds since their milk prices differed too greatly from those of conventional herds (153 yearly records). We also excluded extremely small herds (herd size <1% percentiles; 126 yearly records). In addition, we calculated percentiles for IOFC- cow, IOFC-milk, herd intensity, equity ratio, HM305, relative herd milk price, SCC, calving interval, herd persistency for primiparous cows, herd persistency for multiparous cows and age in days. Of these variables, extreme outliers and records with missing values were excluded (1,887 yearly records). The final dataset included 1,664 herds with 10,499 yearly records.

### Statistical analysis

Using IOFC-cow or IOFC-milk separately as the dependent variable, we developed four linear mixed models. Two models were used to analyse the association between herd economic performance and HLCC; the other two models were used to analyse the association between herd economic performance and HM305. We selected other herd variables as independent variables based on an expected association with IOFC. Those selected herd variables were soil type (sand/other), successor availability (yes/no), equity ratio, herd intensity (kg of milk/ha), milking system (automatic/conventional), use of outsourced heifer rearing (yes/no), relative herd milk price, herd size, expansion rate, SCC and calving interval. We used variance inflation factors to check for multicollinearity between several variables. A year variable was forced onto all models as a fixed effect to account for potential year effects (e.g., absolute milk price differences). A herd variable was entered into the models as a random effect to account for unobserved herd-related heterogeneity (e.g., environment, feed management). To compare the strength of the effect of each independent variable to the dependent variable, we standardized continuous independent variables. Akaike information criterion and backward selection were used to find the best models, which were eventually presented in the results. The conditional R^2^, the marginal R^2^ and the part R^2^ were calculated to describe the variance explained by the entire model, the fixed effects and a single variable, respectively. A Cox test and a J test (31) were used to compare the two non-nested models to investigate whether HM305 or HLCC better explain IOFC. Both tests are used for non-nested hypothesis testing. For example, models A and B are two non-nested models with the same dependent variable. In the non-nested hypothesis testing, model A would have a null hypothesis that the regressors from model B cannot improve the performance of model A. If the null hypothesis of model A is rejected, model B is the “true” model, having an additional explanatory power beyond that contributed by model A. If the null hypothesis of model A is not rejected, model A is the “true” model. The same test can be done for model B to determine whether the regressors from model A can improve the performance of model B.

Data editing and analysis were performed using the Python API for the Spark platform (PySpark) and R version 3.6.3 (32), respectively. Code scripts for the data editing steps, statistical analyses and figure visualizing average herd lactation curve for primiparous cow and multiparous cow can be downloaded at https://github.com/Bovi-analytics/Chen-et-al-2022b.

## Results

**Figure 3:**
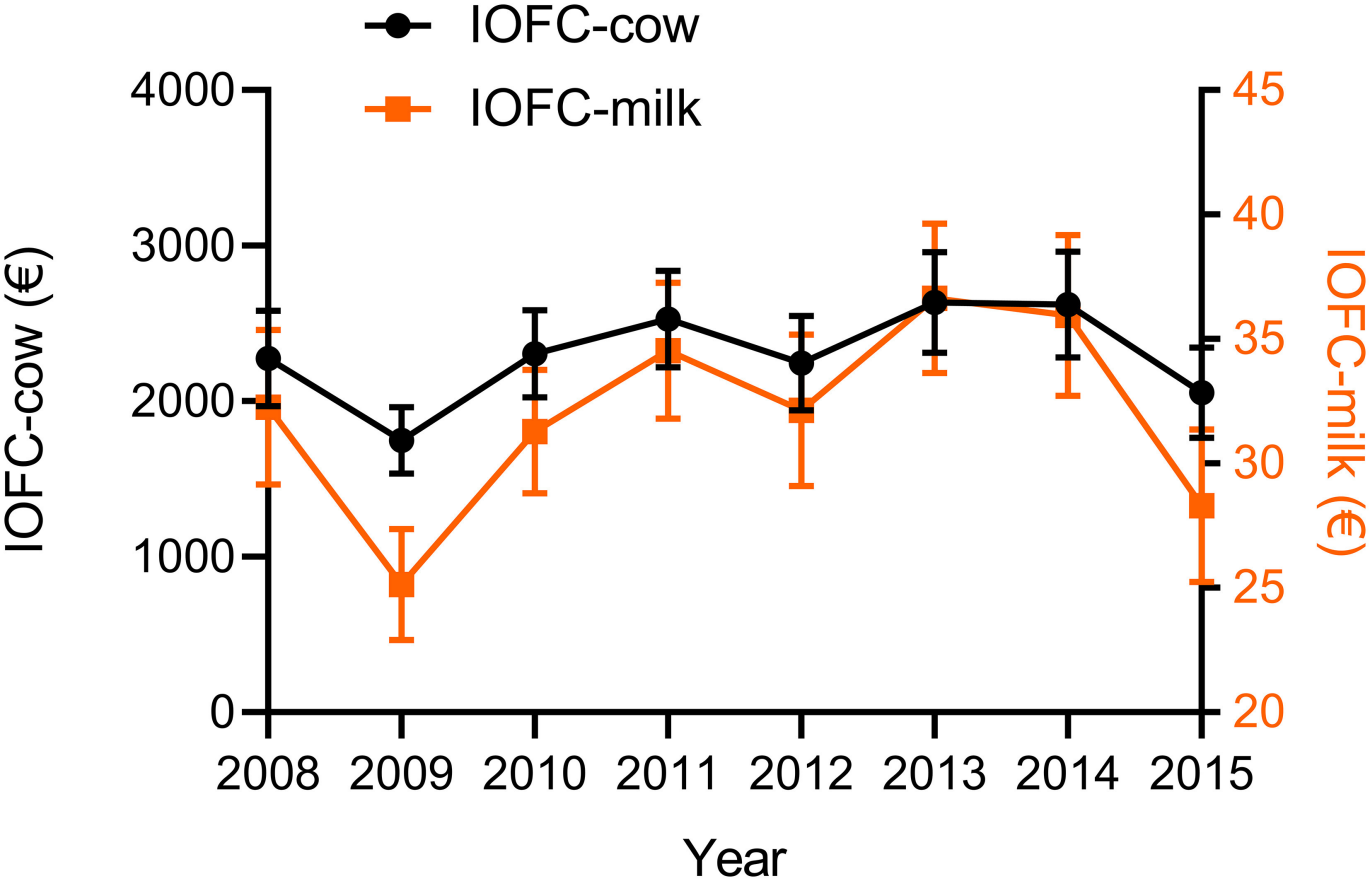
Average income over feed cost per cow (IOFC-cow) and per 100 kg milk (IOFC-milk) for the years 2008 to 2015.

Total feed costs over all farms varied between €20,345 (5%) and €132,519 (95%) per year, with a mean of €63,320. Total revenues likewise varied between €114,589 (5%) and €540,270 (95%) per year, with a mean of €287,787. The descriptive statistics of the continuous variables over all herds and all years are shown in Table 1. The average IOFC-cow was €2,305 (SD = 408) per year, while the average IOFC-milk was €32.1 (SD = 4.6). The same patterns were found in both IOFCs for the years 2008–2015, with the lowest value in the year 2009 and the highest value in the year 2013 (Figure 3). Average herd magnitude, herd time to peak and herd persistency were 34.8 kg (SD = 3.7), 29.6 days (SD = 0.4) and 358 days (SD = 70) for primiparous cows, respectively. Average herd magnitude, herd time to peak and herd persistency were 47.7 kg (SD = 5.3), 22.1 days (SD = 1.3) and 240 days (SD = 33) for multiparous cows, respectively. The average HM305 was 8,686 kg (SD = 899). The average herd intensity was 15,129 kg of milk/ha (SD = 3,841) and the average herd size was 85.3 cows (SD = 43.2).

The results of the final reduced linear mixed models to estimate the associations between the two IOFC definitions and HLCC are presented in Tables 2, 3, respectively.

**Table 2:** Results of the final reduced linear mixed model on the association between income over feed cost per cow (€) and herd lactation curve characteristics (and other herd variables) based on data from 1,664 Dutch herds.

**Variable**		**β**	**S.E**.	***P*-value**
Intercept		2,437.1	5.82	<0.001
Primiparous cows	Magnitude	48.0	3.09	<0.001
	Time to peak yield	1.8	1.85	0.336
	Persistency	13.3	2.44	<0.001
Multiparous cows	Magnitude	154.3	3.72	<0.001
	Time to peak yield	−4.4	1.98	0.027
	Persistency	69.0	2.87	<0.001
Year	2008	Ref^a^		
	2009	−586.3	5.44	<0.001
	2010	−222.2	6.43	<0.001
	2011	85.1	5.78	<0.001
	2012	−241.2	6.32	<0.001
	2013	129.7	6.88	<0.001
	2014	195.2	6.21	<0.001
	2015	−481.8	6.95	<0.001
Herd size		−16.1	3.40	<0.001
Somatic cell counts		−22.9	2.10	<0.001
Herd intensity		−14.7	2.81	<0.001
Calving interval		−21.6	2.12	<0.001
Relative herd milk price		146.4	2.31	<0.001

^a^Ref: used as a reference category.

**Table 3:** Results of the final reduced linear mixed model on the association between income over feed cost per 100 kg milk (€) and herd lactation curve characteristics (and other herd variables) based on data from 1,664 Dutch herds.

**Variable**		**β**	**S.E**.	***P*-value**
Intercept		33.60	0.091	<0.001
Primiparous cows	Magnitude	−0.05	0.036	0.1343
	Time to peak yield	−0.01	0.021	0.5026
	Persistency	−0.13	0.028	<0.001
Multiparous cows	Magnitude	0.07	0.043	0.1174
	Time to peak yield	−0.03	0.023	0.1500
	Persistency	−0.03	0.033	0.3987
Year	2008	Ref^a^		
	2009	−7.54	0.064	<0.001
	2010	−3.58	0.075	<0.001
	2011	0.74	0.069	<0.001
	2012	−2.53	0.074	<0.001
	2013	1.64	0.080	<0.001
	2014	2.07	0.074	<0.001
	2015	−6.51	0.081	<0.001
Soil type	Other soil	Ref		
	Sand soil	0.56	0.085	<0.001
Somatic cell counts		−0.07	0.024	0.003
Equity ratio		0.08	0.028	0.005
Outsourcing heifer rearing	No	Ref		
	Yes	0.61	0.088	<0.001
Herd intensity		−1.21	0.031	<0.001
Calving interval		−0.11	0.025	<0.001
Relative herd milk price		1.88	0.027	<0.001
Expansion rate		0.11	0.018	<0.001
Age in days		0.06	0.025	0.011

^a^Ref: used as a reference category.

All HLCCs were associated (*P* <0.01) with IOFC-cow, except for the herd time to peak yield for primiparous cows (Table 2). Apart from the negative association with herd time to peak yield for multiparous cows, all estimated coefficients were positive, indicating that an increased herd lactation curve characteristic was associated with an increased IOFC-cow. The standardized coefficients indicated that herd magnitude had a larger effect on IOFC-cow for multiparous cows than it did for primiparous cows. Increasing one unit of herd magnitude for multiparous cows and primiparous cows corresponded to a €154.3 and €48 increase in IOFC-cow, respectively (Table 2). In this model, the conditional R^2^ and the marginal R^2^ were 88.9 and 76.9%, respectively. HLCC explained 12.3% variance of IOFC-cow, 86.6% of which was explained by multiparous cows. The top three variables explaining the variance of IOFC-cow were the year, herd magnitude for multiparous cows and relative herd milk price, at 38.2, 10.7 and 7.0% part R^2^, respectively.

The IOFC-milk was negatively associated with herd persistency for primiparous cows (Table 3). A one-unit increase in herd persistency for primiparous cows decreased IOFC-milk by €0.13 (*P* <0.01) on average. In this model, the conditional R^2^ and the marginal R^2^ were 88.7 and 78.9%, respectively. HLCC only explained 0.20% variance of IOFC-milk. The top three variables explaining the variance of IOFC-milk were the year, relative herd milk price and herd intensity, at 53.2, 9.3 and 4.5% part R^2^, respectively.

The results of the final reduced linear mixed models to estimate the associations between the two IOFC definitions and HM305 are presented in Tables 4, 5, respectively.

**Table 4:** Results of the final reduced linear mixed model on the association between income over feed cost per cow (€) and average herd 305-day milk production (and other herd variables) based on data from 1,664 Dutch herds.

**Variable**		**β**	**S.E**.	***P*-value**
Intercept		2,435.2	5.70	<0.001
Average herd 305-day milk production		206.6	2.95	<0.001
Year	2008	Ref^a^		
	2009	−584.5	5.34	<0.001
	2010	−224.3	6.33	<0.001
	2011	86.2	5.73	<0.001
	2012	−248.9	6.25	<0.001
	2013	129.5	6.80	<0.001
	2014	190.92	6.22	<0.001
	2015	−478.1	6.89	<0.001
Herd size		−14.2	3.31	<0.001
Milking system	Conventional	Ref^a^		
	Automatic	21.0	6.31	<0.001
Somatic cell counts		−22.4	2.10	<0.001
Herd intensity		−24.0	2.80	<0.001
Calving interval		−17.8	2.10	<0.001
Relative herd milk price		148.6	2.26	<0.001
Expansion rate		4.84	1.50	0.001

^a^Ref: used as a reference category.

**Table 5:** Results of the final reduced linear mixed model on the association between income over feed cost per 100 kg milk (€) and average herd 305-day milk production (and other herd variables) based on data from 1,664 Dutch herds.

**Variable**		**β**	**S.E**.	***P*-value**
Intercept		33.60	0.091	<0.001
Average herd 305-day milk production		−0.01	0.033	0.700
Year	2008	Ref^a^		
	2009	−7.52	0.064	<0.001
	2010	−3.57	0.075	<0.001
	2011	0.76	0.069	<0.001
	2012	−2.49	0.074	<0.001
	2013	1.65	0.080	<0.001
	2014	2.09	0.073	<0.001
	2015	−6.52	0.081	<0.001
Soil type	Other soil	Ref		
	Sand soil	0.61	0.085	<0.001
Somatic cell counts		−0.08	0.024	0.001
Equity ratio		0.08	0.028	0.008
Outsourcing heifer rearing	No	Ref		
	Yes	0.62	0.088	<0.001
Herd intensity		−1.21	0.031	<0.001
Calving interval		−0.14	0.024	<0.001
Relative herd milk price		1.88	0.027	<0.001
Expansion rate		0.12	0.018	<0.001
Age in days		0.07	0.025	0.006

^a^Ref: used as a reference category.

The final reduced linear mixed model on the association between HM305 and IOFC-cow is shown in Table 4. Increasing one unit of HM305 corresponded to a €206.6 increase in IOFC-cow. In this model, the conditional R^2^ and the marginal R^2^ were 89.6 and 78.7%, respectively. HM305 explained 18.9% variance of IOFC-cow. The top three variables explaining the variance of IOFC-cow were the year, HM305 and relative herd milk price, at 39.5, 18.9 and 7.5% part R^2^, respectively.

The final reduced linear mixed model on the association between HM305 and IOFC-milk is shown in Table 5. HM305 was not associated with IOFC-milk. In this model, the conditional R^2^ and the marginal R^2^ were 88.7 and 78.8%, respectively. HM305 explained 0.03% variance of IOFC-cow. The top three variables explaining the variance of IOFC-milk were again the year, relative herd milk price and herd intensity, at 53.3, 9.1 and 4.2% part R^2^, respectively.

The results of the J test and Cox test are shown in Table 6. For IOFC-cow, there is no difference between the model including HM305 and the model including HLCC. For IOFC-milk, the model including HLCC is significantly better at explaining the variance of IOFC-milk than the model including HM305. However, both HM305 and HLCC variables explained almost no variance at all.

**Table 6:** Results of non-nested hypothesis testing from Cox test and J test.

**Test**	**Comparison** ^**a**^		**Estimate**	**Std**	**Value^b^**	***P*-value**	**Interpretation**
Cox test	IOFC-cow	HLCC–HM305	−576	22.3	−25.8	<0.001	No difference
		HM305–HLCC	−141	25.6	−6.0	<0.001	
	IOFC-milk	HLCC–HM305	−0.9	0.67	−1.38	0.166	HLCC is better than HM305
		HM305–HLCC	−32.2	0.72	−44.5	<0.001	
J test	IOFC-cow	HLCC–HM305	0.9	0.04	24.5	<0.001	No difference
		HM305–HLCC	0.3	0.03	8.8	<0.001	
	IOFC-milk	HLCC–HM305	1.8	1.34	1.3	0.190	HLCC is better than HM305
		HM305–HLCC	1.0	0.12	8.0	<0.001	

^a^IOFC-cow: models for income over feed cost per cow; IOFC-milk: models for income over feed cost per 100 kg milk; HLCC: models include herd lactation curve characteristics; HM305: models include average herd 305-day milk production.

^b^z value for cox test and t value for J test.

All four final multivariable models included variables that showed expected associations with the IOFC outcomes (Tables 2–5). For both IOFC variants, SCC, herd intensity and calving interval were negatively associated, while relative herd milk price was positively associated (*P* < 0.01).

Outsourcing heifer rearing, expansion rate, equity ratio and age in days were positively associated with IOFC-milk (*P* < 0.05). Herd size was negatively associated in both IOFC-cow models, while the milking system was only associated with IOFC-cow when HM305 was present in the model (*P* < 0.01).

## Discussion

The goal of this empirical study was to investigate how HM305 or HLCC are associated with economic performance at herd level, defined as IOFC. We used a unique dataset incorporating 8 years of milk production and accounting data for 1,664 Dutch herds. Accounting data is rarely available on such a large scale (33, 34) and having access to it provided new opportunities to evaluate dairy herd economic performance. In our study, both HM305 and HLCC were associated with IOFC-cow, but they explained approximately the same amount of variance. HLCC is significantly better in explaining the variance of IOFC-milk than HM305. However, both HM305 and HLCC variables explained almost no variance in IOFC-milk at all.

IOFC was chosen as the herd economic performance indicator as the lactation curve is most closely related to milk production and thus milk revenue. In addition, feed costs are between 40 and 60% of the total costs of producing milk (30, 35). Therefore, milk revenues and feed costs seem to be the two economic components that could be most influenced by variations in lactation curves between herds when ignoring other variable costs (such as health and breeding costs). Other studies have, for instance, evaluated gross margin and the milk-to-feed price ratio (22, 36). We chose to focus on IOFC because it is a better measure of profitability in periods of volatility (e.g., fluctuations in milk price) compared, for instance, to the milk-to-feed price ratio (23).

The average IOFC-cow was €2,305 per year, equivalent to €6.22 per day. This value corresponds with previous research on IOFC-cow from similar time periods (37, 38). IOFC-cow was associated with HM305, HLCC (except herd time to peak yield for primiparous cows), year and other herd characteristics (such as relative milk price) (Tables 2, 3). Our findings on the association between IOFC-cow and HM305 correspond with existing literature, as a higher milk yield per cow resulted in a higher IOFC-cow (26). Previously, Laroche et al. (39) had explained that the IOFC-cow depends mainly on milk production per cow. HLCC and HM305 are both indicators that could reflect the herd's production level. That is why they were both highly associated with IOFC-cow. In the current study, HM305 could explain 18.9% variance of IOFC-cow, similar to findings from other studies (40). In the same way, we could explain the HLCCs' association with IOFC-cow by their correlation with HM305. In the HLCC model, herd magnitude was most strongly associated with IOFC-cow among the HLCCs of both parity groups. This was expected, as, of all LCCs, the magnitude has the highest correlation with M305 (19). Herd persistency of both parity groups was positively associated with IOFC-cow although their relative contribution was 2.2–3.6 times smaller than the magnitude. These results correspond with earlier findings (8, 20, 41) and with previous studies also mentioning persistency as an important economic parameter (42, 43). Time to peak yield was least associated with IOFC-cow in our study, supported by a weak phenotypic correlation between the rising rate of milk to the peak yield and M305 (44, 45).

HLCCs for multiparous cows were more strongly associated with IOFC-cow than those for primiparous cows. We expected this finding, since multiparous cows have higher milk production than primiparous cows (46). As multiparous cows generally make up 60–70% of the dairy herd they are thus the main milk suppliers of the herd.

The average IOFC-milk was €33.6, which is in line with previous studies (23, 47). IOFC-milk was not associated with HM305 and HLCC (except for a weak association with herd persistency for primiparous cows). Again, we were not surprised by this finding, as IOFC-milk depends primarily on milk quality payment characteristics (e.g., milk fat and protein) and the cost of concentrates (39). The weak negative association with herd persistency for primiparous cows found in the HLCC model can be explained by the fact that primiparous cows are still growing and need more feed than multiparous cows to produce the same amount of milk (48–50). However, this association was so weak that it only has a small effect compared, for example, to year and relative herd milk price. Other studies using accounting data have illustrated similar challenges in finding economic effects; the hypothesis is that this is due to large heterogeneity between farms and years (22, 34).

Our results indicate that the year effect is most strongly associated with IOFC. The year effect of course reflects the milk price in the Netherlands and we therefore expected, for instance, to see the lowest year effect in 2009 because in that year the milk price was lowest (29). We also found that the relative herd milk price (the price difference in relation to the national raw milk price) was strongly associated with IOFC. This indicates that herds selling milk with a relatively higher milk price due to better components (fat and protein) achieve better economic performance, which is in agreement with previous studies (28, 51). Herd intensity was negatively associated with IOFC, again corresponding with an earlier study (52).

In our study, we defined HLCC by aggregating the individual cow level LCC to a yearly herd level for primiparous and multiparous cows separately. The associations between IOFC and the various HLCCs were deemed logical and interpretable, suggesting that the herd level aggregation was valid. We had expected HLCC to be able to explain more variance in IOFC than HM305 in herd economics since persistent cows are proven to be more profitable in cow level studies (8, 20, 21). However, in the current herd level study, HLCC was not better associated with IOFC than HM305, a finding that we did not expect. There might, however, be logical explanations for this finding. First, the absolute volume of milk production (HM305) is basically the area under the lactation curve. This area consists mainly of the magnitude and the persistency of milk production, and, to a lesser extent, of the time to peak yield. This means that the shape of the curve might essentially be another way to describe the absolute volume of milk production, which is equally captured by M305. A second potential explanation lies in the way LCC is aggregated at herd level. Aggregating HLCC on a calendar year basis is challenging, as individual cow lactation curves often belong to multiple calendar years (17). In our current study, we used the weighted median aggregation method to aggregate HLCC. More sophisticated aggregation methods could probably be used in future studies to improve the aggregation of HLCC. This may result in a more precise HLCC explaining more variance of IOFC than HM305. Potentially, such an improved HLCC might be able to reflect economic variation between herds, irrespective of whether this is defined by IOFC.

In our study, HLCC and HM305 explained a similar variance of IOFC. HLCC is more computationally expensive, while HM305 is more readily available. Potentially, HLCC can be an additional herd indicator, helping farmers and their advisors to evaluate herd lactation when making specific decisions and/or analyses. For instance, when comparing the HLCC of a single herd over several years, the HLCC trends over time may illustrate the genetic improvement of dairy cows for persistency.

## Data availability statement

The original contributions presented in the study are included in the article/supplementary material, further inquiries can be directed to the corresponding author.

## Author contributions

YC: conceptualization, formal analysis, investigation, methodology, software, writing—original draft, and writing—reviewing and editing. MH, MN, and WS: conceptualization, methodology, supervision, and writing—reviewing and editing. JE: methodology and software. All authors contributed to the article and approved the submitted version.

## Funding

YC was financially supported by the Oversea Study Program of Guangzhou Elite Project (GEP).

## Conflict of interest

The authors declare that the research was conducted in the absence of any commercial or financial relationships that could be construed as a potential conflict of interest.

## Publisher's note

All claims expressed in this article are solely those of the authors and do not necessarily represent those of their affiliated organizations, or those of the publisher, the editors and the reviewers. Any product that may be evaluated in this article, or claim that may be made by its manufacturer, is not guaranteed or endorsed by the publisher.

## Abbreviations

HM305: herd's average 305-day milk production
LCC: lactation curve characteristics
HLCC: herd lactation curve characteristics
IOFC: income over feed cost
IOFC-cow: income over feed cost per cow
IOFC-milk: income over feed cost per 100 kg milk
SCC: somatic cell counts.
